# Stable individualized brain computing model informed by spatiotemporal co-activity patterns

**DOI:** 10.1371/journal.pcbi.1013817

**Published:** 2025-12-19

**Authors:** Lan Yang, Jiayu Lu, Xinran Wu, Xi Zhang, Ting Li, Ruiyun Chang, Songjun Peng, Dandan Li, Jie Zhang, Bin Wang

**Affiliations:** 1 The College of Computer Science and Technology, Taiyuan University of Technology, Taiyuan, China; 2 The Institute of Science and Technology for Brain-Inspired Intelligence, Fudan University, Shanghai, China; 3 The College of Software, Taiyuan University of Technology, Taiyuan, China; Vanderbilt University, UNITED STATES OF AMERICA

## Abstract

Accurate simulation of the brain’s intrinsic dynamic activity is essential for understanding human cognition and behavior and developing personalized brain disease therapies. Traditional neurodynamics models depend on structural connectivity to explain the emergence of functional connectivity (FC). However, achieving high-fidelity simulations at the individual level remains challenging, as the models fail to fully capture the brain information. To address these challenges, we introduce the Stable Individualized Brain Computing Model (SI-BCM), a data-driven reverse engineering framework designed to infer spatiotemporal co-activity patterns from fMRI data for simulating whole-brain activity. This model captures the dynamic interactions between brain regions by integrating spatiotemporal dimensional information to extract a stable and shared connectivity pattern, representing the intrinsic functional collaboration pattern of the brain. This connectivity pattern is then used as the core connection weight in the dynamical system. Additionally, the model has a new cost function based on the Phase-Space Association matrix (PSA), enhancing its ability to capture brain activity dynamics. This combination enables the SI-BCM to improve simulation accuracy at the individual level compared to existing models, achieving a correlation coefficient between simulated and empirical FC of 0.87. The SI-BCM also showed enhanced robustness and reliability, and effectively captured brain properties. We found the model sensitively reflected changes in cognitive function, thereby providing valuable insights into the underlying neural mechanisms. Furthermore, the application of SI-BCM in the brain modeling of Alzheimer’s disease (AD) patients substantiated the hypothesis that AD pathogenesis may be due to excessive neuronal excitation. This work establishes a new paradigm for brain network modeling by prioritizing the inference of stable dynamics features from activity data, providing a powerful tool for understanding brain function and pathophysiology.

## Author summary

In our research, we developed a novel brain modeling framework called the Stable Individualized Brain Computing Model (SI-BCM). Our goal was to create a more accurate and personalized tool for simulating brain activity, with a focus on the brain’s own functional dynamics. Traditional models rely on the brain’s structural connectivity, which may not accurately capture individual brain dynamics. Our model employs a reverse-engineering approach, analyzing a person’s brain activity to deduce a spatiotemporal co-activity pattern. This inferred pattern then drives the model to produce realistic brain simulations. We validated the SI-BCM using data from healthy subjects and Alzheimer’s disease (AD) patients. The results showed that our model can simulate brain activity with high accuracy and reliability, capturing individual differences in brain function. Notably, it revealed a correlation between brain activity and cognitive performance, suggesting potential applications in understanding cognitive variations. Furthermore, when applied to AD patients, the model supported the hypothesis that AD may result from excessive neuronal excitation. This work could pave the way for virtual brain modeling, aiding in the exploration of brain mechanisms and the development of personalized treatments for brain diseases. We hope our model will be a valuable tool for both researchers and clinicians in advancing our understanding of the brain.

## Introduction

The brain, a dynamic system of trillion neurons communicating through connections [1], has biophysical behavior shaped by neuronal connectivity patterns and non-linear neural dynamics [2]. Recent researches have built brain computational models by integrating multimodal neuroimaging data using biologically inspired mathematical equations, simulating activity patterns similar to real brain activity [3–5], thereby providing insights into information-processing mechanisms [6]. This deepens the understanding of brain mechanisms, and opens up new approaches to cognitive computation and brain disease research [7–10].

Current predominant whole-brain computational models, like Wilson-Cowan, Kuramoto, Jansen-Rit, and Dynamic Field models, are based on positive - only structural connectivity (SC) from DTI data. These neurodynamics models aim to simulate the mechanisms and interactions of neuronal populations, revealing activity dynamic underlying brain organization [4,6,11]. These models can infer how brain connections generate functional properties [12–15], and explore state-transition mechanisms and pathways [16–20]. They also help study structural connectivity changes and E-I balance disorders’ effects in brain diseases [21–24]. While traditional SC-based models have elucidated some fundamental mechanisms of FC, relying exclusively on SC poses challenges for achieving high-fidelity functional activity simulations at the individual level. Research indicates limitations in using SC to replicate certain complex individual-level functional dynamics [4,7,25,26]. Several potential explanations or causes may underlie this limitation in individual-level brain simulations. One possibility is that the incomplete edges of SC matrices result in a loss of critical brain information [26–28], thereby limiting the accuracy of individual-level simulations. Another possibility is that the SC strength derived from the diffusion tensor imaging (DTI) may not accurately capture the nuanced connectivity patterns required for precise individual-level modeling [29]. Specifically, the anatomical structure parameterized by white matter integrity estimates from diffusion imaging, may introduce potentially false inferences due to limitations in resolving the directionality and functional relevance of fiber tracts [4,17,30]. This discrepancy highlights the challenges in accurately capturing individual variability.

Researchers now focus on functional data-based approaches for whole-brain models. Specifically, functional data shows activity patterns of connections between different brain regions (spatial dimension) and their dynamics over time (temporal dimension) [31,32]. Current studies typically focus on modeling predominantly from either the spatial or temporal perspective. For example, in the spatial dimension, researchers have proposed using empirical Functional Connectivity (FC) to redefine SC strength, or add SC node-pair links during the modeling process [17,26,33–35]. It can infer the “excitatory” or “inhibitory” of white matter bundle from FC, thus reflecting additional information about dynamical mechanisms that are not conveyed by empirical SC [35]. In the temporal dimension, Singh et al. proposed a Mesoscale Individualized Neurodynamics (MINDy) model for fitting time-series data from scratch [29]. Recently, Kashyap et al. leveraged both resting-state and task functional magnetic resonance imaging (fMRI) to construct Sparse Identification of Nonlinear Dynamics (SINDy) by identifying nonlinear dynamics, demonstrating that task-evoked activity is a subset of resting-state dynamics and significantly improving the prediction of measures such as reaction time [36]. Time-series data can capture more dynamic changes in brain activity [37], and then parameterize the model, which can advance the high-resolution characterization of the human connectome [29]. The integration of spatiotemporal information in brain activity analysis can provide a more comprehensive understanding of changes in connection patterns, potentially revealing stable activity patterns with cross-individual functional significance [32,38,39]. However, a key challenge is the explicit extraction of a unified and stable connection pattern that can directly capture the shared information across temporal and spatial perspective.

Unlike traditional neurodynamics models that explore how structure gives rise to function, this study introduces a data-driven reverse engineering framework, the Stable Individualized Brain Computing Model (SI-BCM). We hypothesize that, beyond the static SC, the complex dynamics of brain is dominated by a relatively stable intrinsic spatiotemporal co-activity pattern. This pattern reflects the dynamic collaborative tendencies among brain regions necessary for intrinsic functions. The model’s key innovation is its complete reverse engineering approach: it first infers the spatiotemporal co-activity pattern from fMRI time series data, and then uses this pattern as the core connection weights to drive the whole-brain model, generating highly realistic brain activities. Specifically, it is constructed mainly by extracting the relatively stable and shared connectivity features of brain activity at different time points, representing the computational framework of information-transfer relationships. Among them, the shared information of brain activities, serving as a high-dimensional information matrix, reflects the dynamic changes in brain connectivity over time and space, providing a comprehensive representation of neural activity patterns. Besides, we introduced a new cost function based on Phase-Space Association matrix (PSA), improving the model’s ability to capture dynamic characteristics. The model’s performance is evaluated from global and subnets perspectives using metrics, like similarity, accuracy, robustness, etc. Our Alzheimer’s disease analysis demonstrates altered brain activity patterns may be associated with the overactivity of excitatory neurons. SI-BCM provide a novel method for individualized analysis of aging and degenerative diseases, and offers theoretical support for brain information reconstruction.

## Results

### Study design

**Fig 1:**
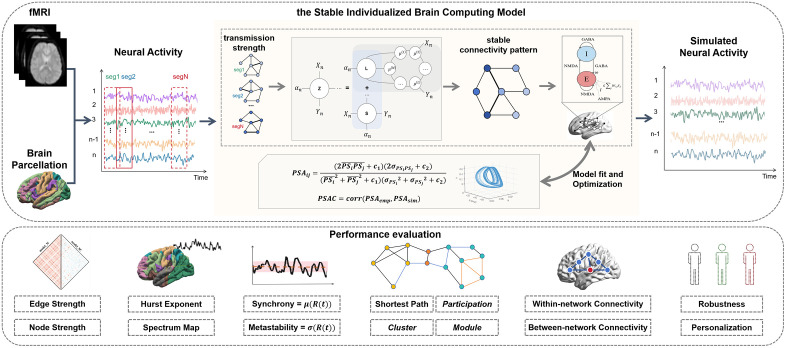
Conceptual overview of the method. The upper part illustrates the construction process of SI-BCM. Firstly, we perform brain parcellation and extract neural activity based on fMRI data. Next, the neural activity is input into the SI-BCM to generate simulated neural activity. The SI-BCM first divides the input neural activities into multiple segments, calculates the transmission strength maps between nodes within each segment, extracts the shared information from these maps, and ultimately constructs the stable connectivity pattern. Subsequently, the model combines the connectivity pattern with the local activity of neuronal populations to achieve brain functional coordination. We also measure the PSA values of the simulated neural activity and the empirical neural activity, and adaptively optimize the parameters of the SI-BCM based on these values to achieve the best simulation (i.e., the maximum PSAC value under the corresponding parameters). Finally, the simulated neural activity generated by the optimal parameters will be output as the result. The lower part presents several indicators for model performance evaluation, comprehensively assessing the model’s performance in simulating brain activity and FC from three perspectives: signals, global networks, and sub-networks.

SI-BCM is a data-driven reverse engineering framework that aims to infer the spatiotemporal co-activity pattern of the brain. This pattern captures the complex dynamics characteristics of an individual’s brain as observed in time series, serving as the core for driving the whole-brain model. Fig 1 illustrates the model’s construction, starting with the extract of preprocessed time series. Unlike traditional models that rely on structural connections, this approach captures instantaneous connection changes by segmenting the time series and calculating dynamic information transmission intensity between brain regions in each segment. A key innovation is the adoption of a decomposition algorithm to extract a shared and stable low-rank connection pattern from these dynamic interactions, which is defined as the spatiotemporal co-activity pattern. This pattern is considered to represent the relatively stable intrinsic collaboration pattern of the brain during the resting state. It is then embedded as core connection weights into a neurodynamics model, such as the dynamic mean field model (see Materials and Methods) or Kuramoto model (see Fig E in S1 Text), to simulate whole-brain neural activity. To ensure the simulated activity accurately reflects the brain’s dynamics characteristics, a PSA is introduced as a cost function. Model parameters are optimized via a genetic algorithm to maximize correlation of empirical and simulated PSA (PSAC). In summary, SI-BCM infers the underlying stable collaborative patterns from observed signals and accurately regenerates brain dynamics.

### Stable individualized brain computing model yielded highly realistic brain activity

The core hypothesis of the reverse engineering framework posits that the spatiotemporal co-activity patterns inferred from fMRI can reproduce the complex dynamics of the brain as connection weights. To validate this hypothesis, we systematically compared the simulation capabilities of the SI-BCM with those of existing models. If the inferred patterns capture the brain’s intrinsic collaborative mechanisms, then the model constructed based on them should generate brain activities that more closely resemble real data across multiple dimensions.

#### Comprehensive signal analysis.

**Fig 2:**
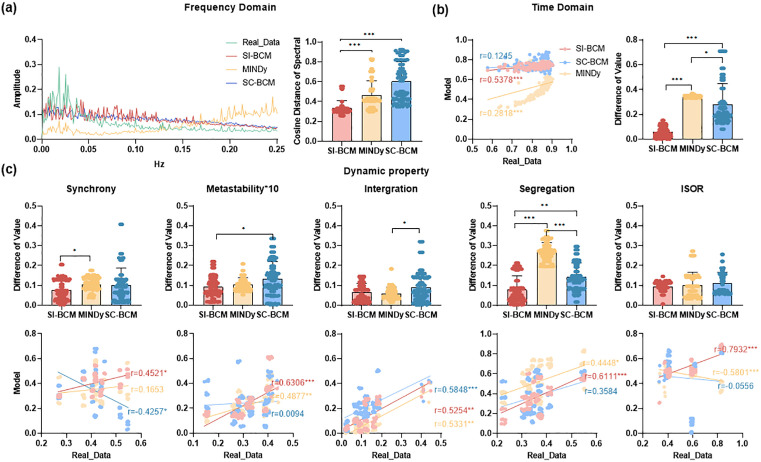
Comparison of real data and model simulations based on various signal analysis metrics. a) Left-side: the group-average Spectrogram of real and simulated data. The green line indicates real data, the red line indicates simulated data generated by SI-BCM, the yellow line indicates simulated data generated by MINDy, and the blue line indicates simulated data generated by SC-BCM. Right-side: the cosine distance of the frequency spectral between individual real and simulated data. b) Left-side: the group-average Hurst Exponent value of real and simulated data. The red, yellow, and blue lines represent linear fit with Pearson correlation coefficient r between SI-BCM, MINDy, SC-BCM, and the real data. Right-side: the difference of Hurst Exponent between individual real and simulated data. c) Upper-side: the difference of dynamic property metrics between real and simulated data. Lower-side: dynamic property metrics of real and simulated data for all subjects. * p < 0.05, **p < 0.01, *** p < 0.001.

Initially, we assessed the simulation accuracy of the three models (SI-BCM, MINDy, and traditional SC constraint-based model (SC-BCM)) in capturing spontaneous resting-state brain activity. We employed adequate metrics to analyze signal aspects, including frequency domain (Spectrogram), time domain (Hurst Exponent), and dynamic property metrics (Synchrony, Metastability, Integration, Segregation, ISOR). In the simulation of real data, while the average spectral curves of SI-BCM and SC-BCM appear similar, SI-BCM consistently excels in accurately reproducing individual spectral shapes, evidenced by significantly lower cosine distances (Fig 2a). The cosine distance between the simulated and real data spectra quantifies differences in their shapes, disregarding amplitude variations. It emphasizes consistency in the distribution patterns of peaks and valleys. A smaller value signifies greater similarity in spectrum shape. This demonstrates SI-BCM’s ability to capture both group-level frequency-domain characteristics and individual spectral patterns. Conversely, the MINDy model exhibits substantial discrepancies from real data in both average shape and individual accuracy. SI-BCM simulated the complexity of the real data more closely than both MINDy and SC-BCM in time-domain measures (Fig 2b). This indicates that the reverse inference model better preserves the dynamic characteristics of long-range correlations in brain activity. Fig 2c illustrates the differences in the dynamic property values between simulated data produced by three models utilizing optimal model parameters and the real data. The results indicate that the inferred co-activity patterns can more effectively drive the model to generate signals exhibiting the dynamic characteristics of real brain activity, compared to traditional approaches relying on structural connections or pure time-series fitting.

#### Global network properties assessment.

**Fig 3:**
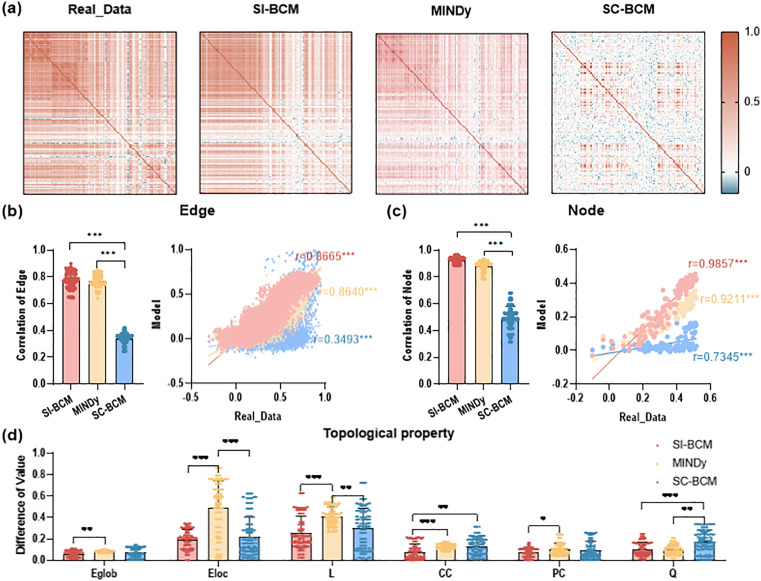
Comparison of real data and model simulations based on various network metrics. a) Diagram of empirical FC and simulated FC by the three models. b) Left-side: the Pearson correlation of the edge FC between real and simulated data. Right-side: edge FC value of real and simulated data for a random subject. The red, yellow, and blue lines represent linear fits with Pearson correlation coefficient r. c) Left-side: the Pearson correlation of the node FC strength between real and simulated data. Right-side: node FC strength value of real and simulated data for a random subject. The red, yellow, and blue lines represent linear fits with Pearson correlation coefficient r. d) The difference of topological property metrics between real and simulated data. Eglob: global efficiency, Eloc: local efficiency, L: characteristic path length, CC: clustering coefficient, PC: participation coefficient, Q: modularity. * p < 0.05, **p < 0.01, *** p < 0.001.

Subsequently, we evaluated the metrics of the three models in capturing the properties of FC: static pairwise FC (edge FC), static node-level average FC (node FC), and topological property metrics (global efficiency, local efficiency, characteristic path length, clustering coefficient, participation coefficient, modularity). SI-BCM is more closely with real data in terms of network structure and strength (Fig 3a and 3b). Fig 3c demonstrates that SI-BCM exhibits superior accuracy than other models in capturing topological properties. This indicates that the pattern inferred by our model is not merely mathematical abstractions. In fact, it encodes the organizational principles of the brain’s functional network, and can reproduce its global and stable connection architecture with high fidelity.

#### Sub-networks simulation performance evaluation.

**Fig 4:**
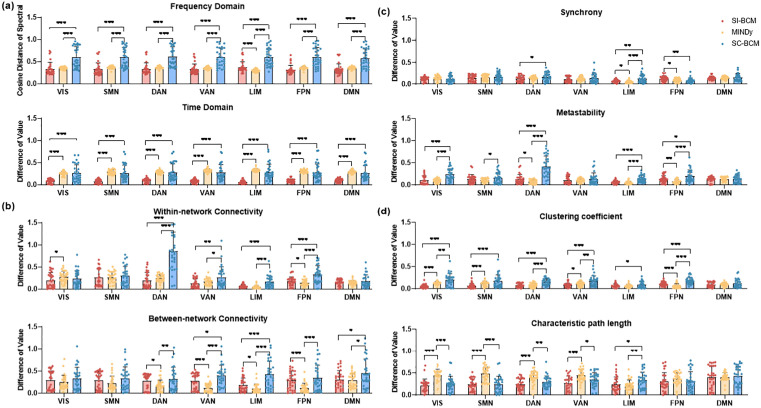
The measurement of the simulation performance of three models for different subnets. a) and c) The simulation performance of different subnets is measured by measuring the similarity between real and simulated signals. b) and d) The simulation performance of different subnets is measured by measuring the similarity between the evaluation metrics of the signal and network level of real data and simulated data. VIS: visual network, SMN: somatomotor network, DAN: dorsal attention network, VAN: salience/ventral attention network, LIM: limbic network, FPN: frontoparietal control network, DMN: default mode network. * p < 0.05, **p < 0.01, *** p < 0.001.

Additionally, we evaluate the simulation results generated by the three models in resting-state networks. Fig 4a illustrates that the signals simulated by SI-BCM are closer to the real data in the time-frequency domain in each sub-network, except for LIM. The simulation results of SI-BCM outperform SC-BCM regarding dynamic properties in each subnetwork, and are comparable to MINDy(Fig 4c). SI-BCM is more effective than MINDy in capturing within-network connectivity strength and topological properties for network analysis, but it is less effective in capturing between-network connectivity strength (Fig 4b and 4d). This result reveals that the pattern we inferred is adept at characterizing the stable collaborative relationships within the brain’s functionally specialized subnetworks, which might be the key to its ability to improve the accuracy of individualized simulations.

#### Model performance comparison.

We provide many model inputs and their corresponding simulation performance outcomes (similarity between empirical FC and simulated FC (FC_cor), similarity between empirical dynamic functional connectivity (dFC) and simulated dFC (dFC_cor), Kolmogorov-Smirnov (KS) distance between empirical functional connectivity dynamics (FCD) and simulated FCD (FCD_KS)). The results of SI-BCM are superior to those of the existing models (Table 1). All results in the table were rounded to two decimal places to ensure consistency and clarity in data presentation. This study also examined a range of metrics, beyond merely these three standard measurements. This result suggests that the connection pattern more effectively drives large-scale brain network dynamics than either empirical structural connections or time series fitting. This provides preliminary and robust support for the core hypothesis that a stable collaborative pattern in brain activity exists and can be extracted to dominate its dynamics characteristics.

**Table 1:** Comparison with existing models.

Model	Input	FC_cor (↑)	dFC_cor (↑)	FCD_KS (↓)
Dynamic MFM [40]	empirical SC	0.46	0.44	0.73
pMFM [41]	empirical SC	0.56	0.60	0.5
DTB [34]	Add links to empirical SC	0.82	0.74	0.40
Kuramoto Model [35]	functional connectivity-informed SC	0.86	0.72	0.42
MINDy[29]	fMRI	0.86	0.76	0.40
**SI-BCM**	**fMRI**	**0.87**	**0.79**	**0.39**

### Stable individualized brain computing model is generalized, personalized, and robust

**Fig 5:**
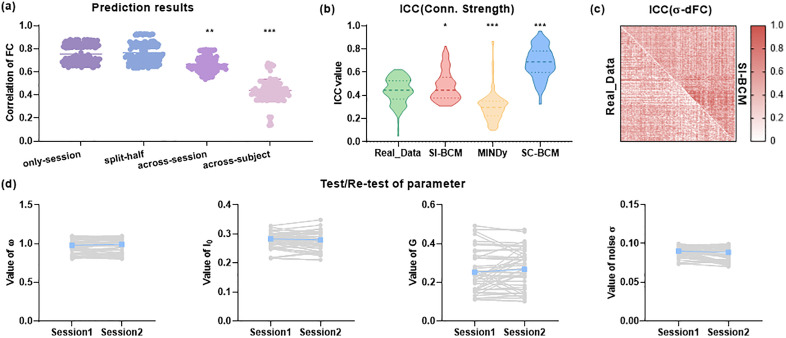
The generalization, personalization, and robustness of SI-BCM. a) Comparison of predictive similarity of SI-BCM in different cases. Split-half (within-subject): in the case of the same subject and session, predict the similarity between the first half and the second half. Across-session: comparison of predictive similarity between sessions of the same subject. Across-subject: comparison of predictive similarity within and between subjects. b) Evaluate the test-retest reliability of the connection strength between real data and the model. c) Comparison of reliability between SI-BCM and real data for predicting each subject’s σ-dFC for each pair of brain regions. d) The distribution of optimal model parameter values of SI-BCM for the test-retest sessions. The blue line indicates the group-mean. * p < 0.05, **p < 0.01, *** p < 0.001.

A good model must demonstrate substantial generalization ability. To rigorously evaluate our model’s generalization ability, we conducted a series of independent simulation performance analyses across different collection scenarios (Fig 5a). These scenarios are specifically defined as follows: 1) Split-half (within-subject): For the same subject and session, we calculated the similarity between the simulated values based on the first half of the session signal and the simulated values of the second half of the signal. This allows us to evaluate the stability and consistency of the model within a single session. 2) Across-session: For the same subject and different sessions, we calculated the similarity of the simulated values between session signals of the same subject on different days, with an interval of 1 day between sessions. This helps us evaluate the model’s generalization ability for the same subject over time. All analyses indicated that SI-BCM exhibits a degree of generalization for each subject, as evidenced by the results of the Split-half and Across-session assessments.

To assess the model’s sensitivity to external interference, we examined the stability of the optimal model parameters within individuals and across scanning sessions. While the parameters exhibited good consistency across sessions (Fig 5d), the standard deviation of the differences in the global constant G was notably larger than that of other parameters. This is significant, as it suggests that despite the relative stability of individual brain connection patterns over short time spans, the instability of G likely arises from uncontrollable external factors during scanning sessions. These factors include environmental noise, minor variations in scanning protocols, or fluctuations in the subject’s physiological state on the scanning day. Such variability forces the model to adjust the global constant, potentially impacting its generalization performance for the same subject over time.

Moreover, We compared the simulation similarities between subjects on the same day, quantifying the model’s variability ability to capture inter-subject. The results show that inter-subject simulation similarities are significantly lower than inter-session predictive similarities within the same subject (Across-subject in Fig 5a). This indicates that SI-BCM can effectively capture the unique differences between individual brains. This directly indicates that the patterns extracted by SI-BCM capture individual-specific rather than group-general neural features.

For the robustness of SI-BCM, we analyzed the test-retest reliability of the network, including both static and dynamic networks. These data are derived from resting-state scans of human subjects over two separate days. We also identified the optimal model parameter values for each day. Here, the σ-dFC metric represents the temporal variability of dynamic functional connectivity, quantified as the standard deviation of time-evolving correlation patterns. This measure can also be interpreted as capturing the signal power of the moving-correlation time series. Empirical evidence has consistently validated σ-dFC as a robust and reliable metric for assessing dynamic functional connectivity characteristics [42]. Firstly, the optimal model parameters over two days exhibited low variability (Fig 5d), with p-values from paired t-tests ranging between 0.16 and 0.20. This indicated that the model parameters remained stable across different testing sessions. Secondly, the simulated results for test-retest sessions are more stable than real data, both in static and dynamic networks (Fig 5b and 5c), indicating that our model can more accurately capture the inherent stability of brain functional connectivity patterns. These findings confirm that stable co-activity pattern is a reproducible and reliable core component in the individual brain functional architecture. In comparison to MINDy, the outcomes of SI-BCM more closely with the real data (Fig 5b). Compared to MINDy, the averaged ICC value of SI - BCM were about 0.18 higher on, indicating a higher agreement with real data. In comparison to SC-BCM, the ICC values range between 0.4 and 0.6. The outcomes of SI-BCM more accurately reflect the variations between cross-sessions (Fig 5b) and more closely with real-world situations. These comparison results further highlight the advantages of our model. Furthermore, the individual differences in the σ-dFC of simulated models exhibit a high degree of stability when compared to real data across most region pairs (Fig 5c).

### Combining cost function PSAC generated more realistic brain dynamics

**Fig 6:**
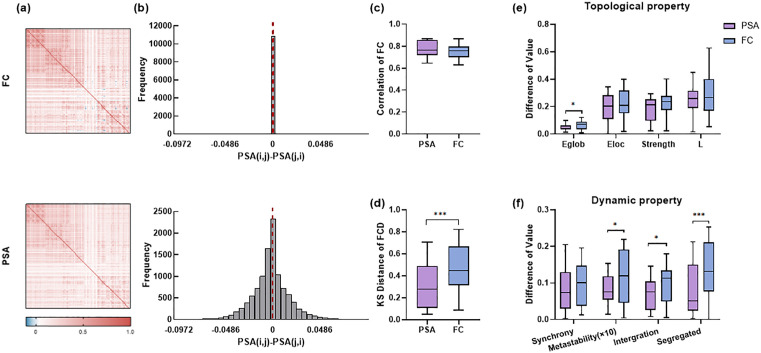
Importance of cost function PSAC for generating realistic brain dynamics. a) Diagram of FC (upper-side) and PSA (lower-side) of real data. b) Quantitative analysis of the asymmetry in the FC (upper-side) and PSA (lower-side) is conducted using a histogram depicting the distribution of differences between the upper and lower triangular elements. c) Pearson correlation of FC between real data and simulated data obtained utilizing two cost functions. d) The KS distance of FCD between real data and simulated data obtained utilizing two cost functions. e) The difference in topological property metrics between real data and simulated data obtained utilizing two cost functions. f) The difference of dynamic property metrics between real data and simulated data obtained utilizing two cost functions. Eglob: global efficiency, Eloc: local efficiency, L: characteristic path length, Syn: synchrony, Meta: metastability(*10), Inter: intergration, Segre: segregated. * p < 0.05, **p < 0.01, *** p < 0.001.

We conducted multiple analyses to explore the significance of cost functions in the generation of brain activity. Initially, as shown in Fig 6a, the Phase-Space Association matrix (PSA) is asymmetric, which forms a contrast with the symmetric properties of the FC matrix. To quantify PSA asymmetry, we compared its upper and lower triangular elements in Fig 6b. The distribution of differences among multiple data points deviates from zero in the PSA, providing definitive evidence of the PSA matrix’s statistical asymmetry. This suggests that the dynamic influence of brain region i on region j may not equal that of j on i. And we also examined the results of employing cost functions that correspond to PSA and FC for the generation of static FC and FCD. Fig 6C and 6D demonstrate that PSAC is more effective in preserving the dynamic information of brain activity. Additionally, we analyzed the topological properties (Fig 6E) and dynamic properties (Fig 6F), revealing that the brain activity produced by PSAC more closely with real data in terms of dynamics compared to that generated by correlation of empirical and simulated FC (FCC). This finding suggests that the PSAC cost function directs the optimization process to align the inferred connection patterns with the phase-space trajectories of observed brain activity, thereby enhancing the likelihood that the inferred patterns can generate brain activity within the empirically observed dynamic range. In other words, the PSA approach ensures that the inferred patterns capture not merely a static snapshot but rather a driver containing rich dynamics information.

### The spatiotemporal co-activity pattern exhibits biological plausibility

**Fig 7:**
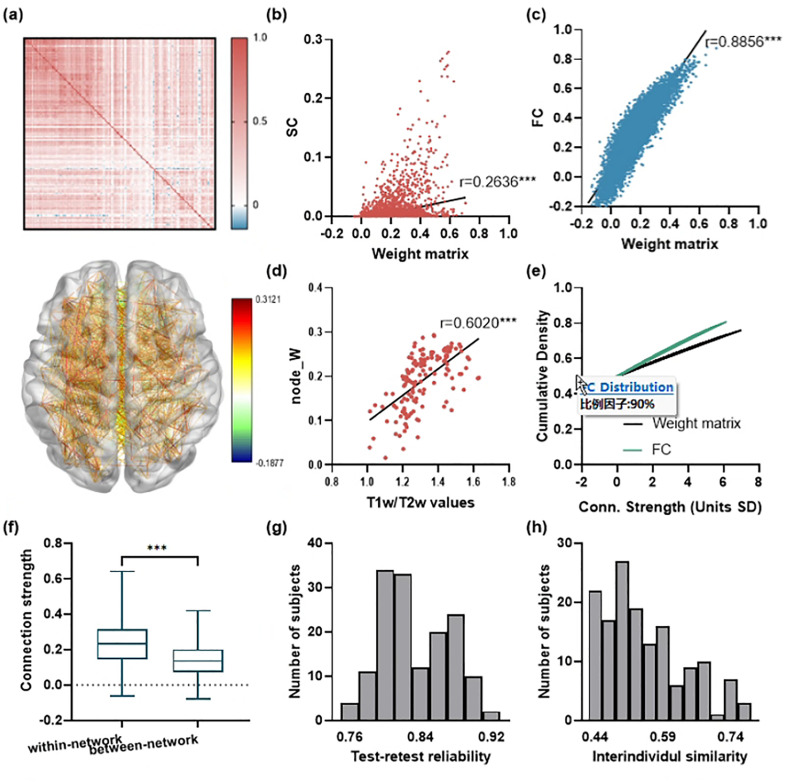
Characterization of the inferred spatiotemporal co-activity pattern. a) upper-side: The inferred spatiotemporal co-activity pattern matrix for a representative subject. lower-side: thresholded anatomical projection (positive connections ≥20% max non-recurrent magnitude and negative connections with magnitude ≥5%). b) Correlation between edge weights of spatiotemporal co-activity pattern and SC. c) Correlation between edge weights of spatiotemporal co-activity pattern and FC. d) Correlations between T1w/T2w values and node strength of spatiotemporal co-activity pattern. e) The weight distribution of spatiotemporal co-activity pattern demonstrates sparser connectivity than FC. f) Comparison of within-network versus between-network connection strengths in spatiotemporal co-activity pattern. g) Test-retest reliability of spatiotemporal co-activity pattern across scanning sessions. h) Inter-individual similarity of spatiotemporal co-activity pattern.

We conducted a systematic analysis to validate the inferred spatiotemporal co-activity pattern. The matrix (Fig 7a) displayed a distinct modular structure, and the corresponding brain map confirmed its spatial distribution. Notably, the matrix showed significant correlations with both SC and FC (Fig 7b, c; SC: r = 0.2636, p < 0.001; FC: r = 0.8856, p < 0.001), indicating it reflects a stable interaction between anatomical and functional connections. Further biological validation revealed a significant correlation between the matrix’s node strength and myelination degree (Fig 7d; r = 0.6020, p < 0.001), supporting its structural basis. Additionally, we quantified the sparsity of the matrix using the kurtosis found that there was a significant difference between this weight matrix and the rsFC, and the former had higher sparsity (Fig 7e). The kurtosis of the weight matrix (37.9 ± 6.3) of each subject greatly exceeded that of the rsFC (19.5 ± 5.1). Network organization analysis showed that connection strength within the matrix network was significantly higher than between networks (Fig 7f; p < 0.001), aligning with known brain functional architecture. The present study also demonstrated the individual-specific nature of the spatiotemporal co-activity pattern. Test-retest reliability analysis revealed high temporal stability (mean ICC = 0.8433, Fig 7g), while the inter-individual similarity analysis confirmed the matrix’s ability to effectively distinguish between different individuals (Fig 7h). In sum, these findings indicate that the spatiotemporal co-activity pattern is a biologically meaningful computational entity, which not only preserves the key features of the brain’s organization but also exhibits marked individual specificity and temporal consistency.

### The relationship between brain activity and cognitive performance

**Fig 8:**
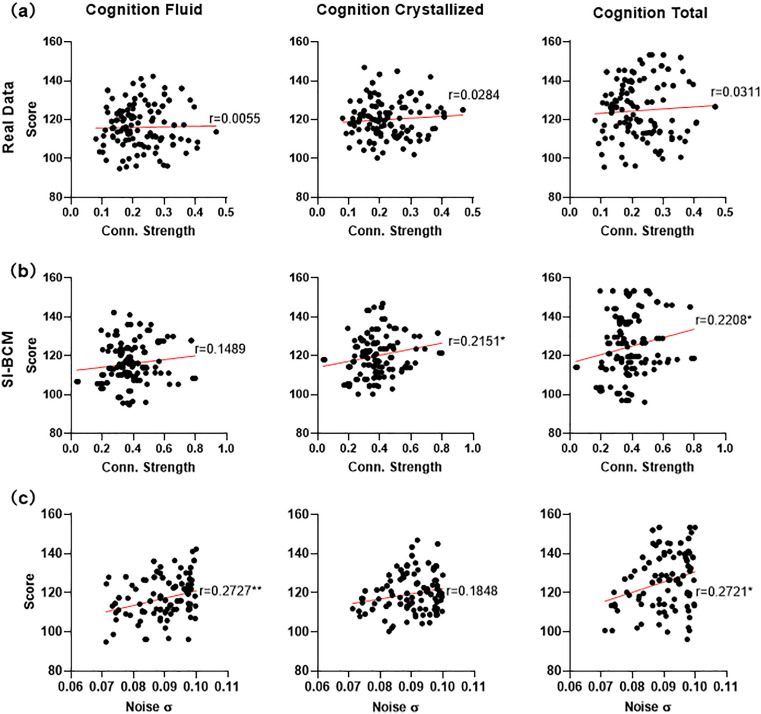
The correlation between brain activity and cognitive performance. a) The correlation between cognitive performance and averaged connection strength of empirical FC. b) The correlation between cognitive performance and averaged connection strength of simulated FC. c) The correlation between cognitive performance of healthy subjects and noise σ. * p < 0.05, **p < 0.01, *** p < 0.001.

A linear regression analysis was conducted to examine the relationship between cognitive performance and brain activity, specifically focusing on the connection strength of both empirical and simulated FC. Cognitive performance is represented by cognitive intelligence and its composite score, including both fluid and crystallized intelligence. Fig 8b shows a significant positive correlation between cognitive performance and simulated brain activity, which is absent in real data (Fig 8a). This may be attributed to the fact that the stable connectivity pattern contains more detailed neural connectivity patterns and neurotransmitter activities, which are closely related to information processing efficiency. These elements enable the model to more accurately capture activity associated with the brain’s cognitive state. Specific neural connectivity patterns have been proven to facilitate efficient information transmission between brain regions, which is crucial for cognitive tasks [43]. The model enhances its ability to reflect cognitive performance by inputting a stable connectivity pattern.

The model parameters denote the dynamic properties of neuronal connections [6,44]. The properties affect neuronal interactions and information transmission, thereby impacting brain activity and functional performance. To examine the relationship between model parameters and brain states, we conducted linear regression analysis correlating the model parameters with the cognitive performance of the subjects. Fig 8c illustrates a notable positive correlation between the fluid intelligence scores of healthy subjects and the model’s noise parameter. The noise present in this model differs from conventional interference as it directly influences neuronal activity and neural information processing. Specifically, an increase in noise strength makes neurons generate more discharges.

### Simulation analysis based on Alzheimer’s disease

**Fig 9:**
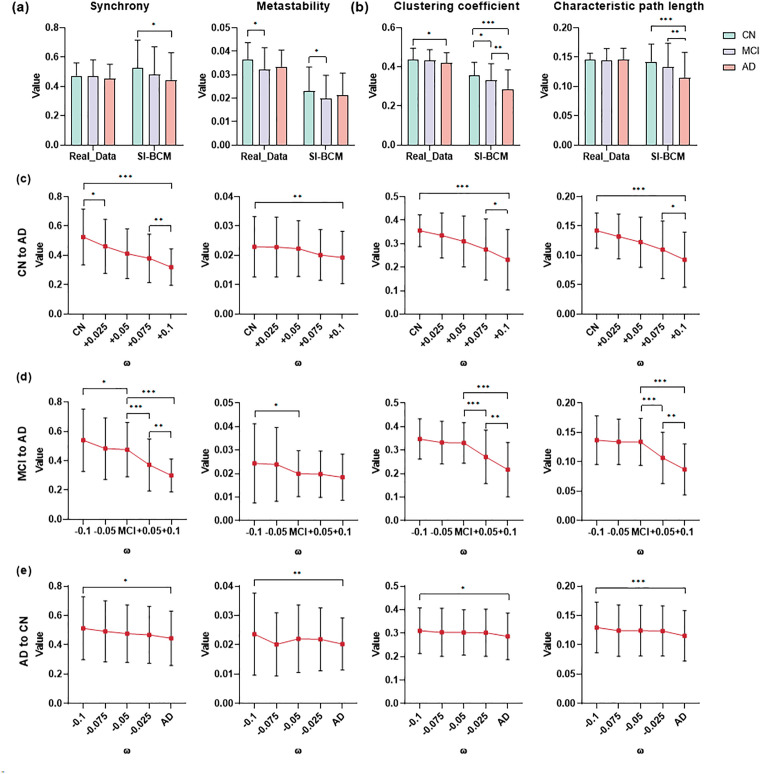
Simulation analysis based on Alzheimer’s disease. a) Comparison dynamic property of simulated data and real data. b) Comparison topological property of simulated data and real data. c) Exploring the changes of dynamic property and topological property of brain activity by continuously increasing the value of the recurrent connection strength ω for the CN model. d) Exploring the changes of dynamic property and topological property of brain activity by continuously adjusting the value of the recurrent connection strength ω for the MCI model. e) Exploring the changes of dynamic property and topological property of brain activity by continuously decreasing the value of the recurrent connection strength ω for the AD model. * p < 0.05, **p < 0.01, *** p < 0.001.

This study employs disease data to conduct a validation analysis aimed at assessing the ability of SI-BCM to replicate the characteristics of the disease. We reproduced the typical changing trends in brain activity indicators (Fig 9a, b). More importantly, we also observed systematic fluctuations in the underlying model parameters that govern these dynamic changes.

In studying the model’s impact on disease progression, we focused on the recurrent connection strength ω. For Alzheimer’s disease, neuronal hyperexcitability is a key pathological feature. Changes in recurrent connection strength can directly affect neuronal excitability, aligning with the neurobiological mechanisms of the disease. When building the SI-BCM, the recurrent connection strength, as a critical parameter, is sensitive to the dynamic changes in brain networks across different disease stages. These changes are consistent with alterations in neuronal activity patterns caused by neurotransmitter level changes and synaptic dysfunction in Alzheimer’s patients, enabling the model to effectively capture the disease’s evolution characteristics. Fig C in S1 Text shows the optimal parameter distributions for SI-BCM in cognitively normal (CN), mild cognitive impairment (MCI), and Alzheimer’s Disease (AD), where the recurrent connection strength appears difference. During the simulation process, we adjusted the recurrent connection strength to simulate changes in brain activity across different disease stages. First, by increasing the recurrent excitation strength in the CN model, we simulated the progression towards a hyperexcitable AD-like state (Fig 9c). Conversely, reducing ω in the AD model simulated a theoretical therapeutic intervention aimed at alleviating hyperexcitation, partially reversing the dynamics towards the CN state (Fig 9e). Adjusting ω in the MCI model demonstrated its role in steering the network towards health or disease (Fig 9d). In all manipulations, the model consistently showed that disease progression significantly decreased synchrony, metastability, clustering coefficient, and characteristic path length. These changes aligned with brain network dysfunctions observed in the actual disease, indirectly reflecting the impact of disease-specific mechanisms on brain network function. When we adjusted the parameters of the SC-BCM model using the same method, although the four metrics also showed a downward trend (Fig D in S1 Text), its ability to capture these characteristic changes was inferior to the SI-BCM. Therefore, the SI-BCM simulation framework not only serves as a modeling tool, but also enables the quantitative assessment of system parameters. By inferring parameter changes in a reverse manner, the model provides computational evidence supporting the pathological hypothesis of neuronal hyperexcitability in AD.

## Discussion

This study introduces the Stable Individualized Brain Computational Model (SI-BCM), a data-driven reverse engineering framework. The core of this framework is the reverse inference of a stable spatiotemporal co-activity pattern from time series, which drives whole-brain dynamics. Our findings demonstrate that this framework can accurately reproduce personalized brain activity patterns, while the inferred pattern itself exhibits individual specificity, stability, and biological plausibility. Crucially, by modulating the model’s parameters, this framework can offer computational insights into the neural mechanisms underlying cognitive differences and brain disorders, such as Alzheimer’s disease.

### SI-BCM can generate realistic spontaneous brain dynamics

SI-BCM attempts to address the issue that FC cannot predict how to relate the property of experimental operations to observed brain activity changes. Its aim differs from true generative whole-brain models, such as those implemented in the Virtual Brain Twin, which simulate multimodal data using anatomical priors [7]. Although those generative models link directly to biophysically interpretable parameters and can reproduce diverse paradigms, they are constrained by incomplete individual structural connectivity and by a small set of free parameters, limitations that reduce their fidelity in capturing individual functional dynamics. By contrast, our model employs nonlinear dynamics theory to describe the interactions of real inter-neuronal connections and captures the physical processes of brain activity in both the temporal and spatial dimensions, thus providing a richer view of brain mechanisms than FC. SI-BCM uses time series for fitting from scratch, rather than measures such as diffusion imaging. While these structure-based approaches provide a direct link to anatomy and enhanced biological realism [4], our model directly derives collaboration rules from functional dynamics alone. This approach significantly enhances simulation accuracy at the individual level, enabling a more precise depiction of the extended patterns of spatiotemporal brain dynamics. Suárez et al. believe that most existing models have an incomplete correspondence between SC and FC [45]. They also suggest combining one or more signals to redistribute weights for connectivity, which could result in higher fidelity and more detailed information about the brain’s spatial [45]. SI-BCM takes advantage of this and simulates signals that are closer to real data than SC-BCM’s, especially in reflecting the brain’s complexity and functional state. Concurrently, the greater flexibility of the functional data enabled the SI-BCM to more effectively capture the brain’s capacity for independent information processing and its ability to integrate multiple functions and generate coordinated neural oscillations. This doesn’t mean that traditional models are ineffective, rather, they may be constrained in the study of some scientific issues.

Particularly, MINDy and SINDy also use the fMRI for fitting from scratch, and is able to generate valid long-term patterns of brain dynamics [29,36]. While SI-BCM, SINDy and MINDy have similar general frameworks, the details inside are quite different. The innovation of SI-BCM is its ability to abstract a stable spatial pattern from dynamic interactions, rather than simply fitting time series. This pattern, serving as a medium for cross-temporal information, enhances the model’s capacity to capture long-term dependencies and the intricate nature of brain activities, thereby improving its generalization and robustness. SINDy automatically identifies concise and interpretable differential equations from observational data through sparse regression, thereby revealing the dominant dynamic laws behind the system [36]. But our model is specifically designed to infer the global stable connectivity architecture that orchestrates these regional dynamics into a coherent whole-brain pattern. SI-BCM provides a network-level understanding of brain organization, complementing the equation-level insights offered by SINDy. Besides, MINDy ignores the description of inhibitory neuronal firing processes, but SI-BCM is unrestrained in describing the relationship between the activities of neural populations. This relationship can be described by the firing processes of multiple neuron classes. In computational models, even small local modulations of excitation-inhibition balance can substantially change the organization of functional interactions [42,45,46]. So SI-BCM is better than MINDy for capturing brain information processing. Specifically, SI-BCM captures individual differences better than MINDy. In comparisons of metrics analyses, SI-BCM is also better than MINDy in capturing both the complexity of brain activity and the ability to interact with brain information. This is further evidence that SI-BCM can simulate more brain effective information.

In summary, the SI-BCM provides a new modeling paradigm. On one hand, it extracts stable pattern from functional data that reflect the brain’s complex dynamics and information processing capabilities, providing interpretable insights into brain function. On the other hand, it can be used to predict the spatiotemporal dynamics of brain activity.

### The spatiotemporal co-activity pattern has a biological significance

The spatiotemporal co-activity pattern identified in this study is a computational entity with clear biological significance. This pattern is not only related to SC and FC but also significantly different. Furthermore, they spontaneously exhibit the organizational principle of brain functional modularity. These evidences suggest that the spatiotemporal co-activity pattern captures a dynamic architecture between structure and function. Importantly, this architecture is fundamentally different from effective connectivity generated by DCM [47], which aims to characterize the instantaneous and directional causal influence between brain regions in a state-dependent manner. In contrast, the spatiotemporal co-activity pattern does not describe the directional information flow at a specific moment, but rather a stable and undirected collaborative infrastructure extracted from cross-temporal dynamic interactions. This represents a stable collaborative potential or tendency between brain regions, which provides a basis for the emergence of various instantaneous effective connectivity patterns. Moreover, some studies have rigorously demonstrated that most of the temporal fluctuations observed in functional connectivity can be explained by the sampling variability of a stationary linear process rather than genuine non - stationary, discrete state transitions [48]. The stable spatiotemporal co-activity patterns we inferred can be regarded as the core engine generating a continuous and smooth dynamic process. And our model has also successfully captured the core dynamic characteristics of real brain signals. Consequently, our findings offer a new computational perspective for understanding the interplay among brain structure, function, and dynamics. The spatiotemporal co-activity patterns serve as a key intermediary connecting these elements, forming a stable functional architecture that supports and constrains dynamic information flows.

### PSA gives more consideration to dynamics property

The new proposed PSA matrix has been shown to significantly enhance the characterization of effective connectivity between brain regions in resting-state FC. Rabinovich et al. proposed that sequential transient brain dynamics can be seen as the result of sequential switching of observable metastable states [49]. In this context, phase space refers to a multi-dimensional space, and each dimension represents a variable describing the system’s state. For brain dynamics, phase space can represent the activity levels of different brain regions or the rates of change in these activities. The phase space trajectory is used to describe the path taken by the system state during transitions between different metastable states [50]. The PSA matrix considers the degree of brain activity switching between two brain regions, making it very suitable for understanding and describing neural dynamic systems. Unlike the elements of the correlation matrix, the PSA matrix can describe asymmetric connection strengths and gives more consideration to dynamics properties. In optimizing SI-BCM, PSA serves as a critical cost function. Its asymmetry allows it to capture dynamic directional information overlooked by FC, guiding the model to optimize connection patterns that produce a more realistic dynamic range. This underscores that employing dynamic features like PSA as reverse engineering targets more effectively reveals the brain’s intrinsic dynamics than aligning static statistics like FC.

### SI-BCM can explore the relationship between brain activity and behavior

In this work, we found that brain activity was positively correlated with general intelligence. Compared to the empirical data, the simulated data more clearly demonstrated this correlation. This finding illustrates that SI-BCM can be used to explain individual differences in cognitive performance. This also suggests that subjects’ ability to answer questions correctly is related to their internal neuronal dynamics. There are some works that have explored the specific link between intelligence and neurodynamics [37,51–53]. Correspondingly, our model more saliently represents the neuronal dynamics within the brain, improving the precision of the analysis. In addition, this indirectly shows that our model can help remove some of the noise during the signal acquisition process, making the key signals more outstanding for subsequent research and analysis.

In this study, we found a significant positive correlation between fluid intelligence and parameter noise in the resting-state brain activity of healthy subjects. This means that higher values of parameter noise are associated with higher fluid intelligence scores and better performance on cognitive task. Parametric noise in SI-BCM helps to stimulate brain neurons, and has a certain activating effect on the brain [54]. Specifically, the higher value of parameter noise, the faster the fluctuations in brain activity. Faster fluctuations of brain activity correspond to the information processing ability of neurons being superior, and higher fluid intelligence score [55].

### AD may be caused by excessive neuronal excitation

Utilizing the SI-BCM, we successfully replicated the key characteristics of disease states with high fidelity. The model accurately captures typical trends in brain activity metrics such as synchronicity, metastability, clustering coefficients, and feature path lengths in different disease states. These findings, supported by previous research, have established the importance of these metrics in characterizing brain function and dysfunction [56,57]. Specifically, with the development of AD, the global synchrony, the clustering coefficient, and the characteristic path length of MCI and AD were decreased, and the global metastability showed a tendency to decrease and then slightly increase. These results also illustrate that SI-BCM is a robust tool for brain disease modeling. Its ability to accurately reflect the typical trends observed in empirical data suggests that it can serve as a valuable platform for further investigation into the neural mechanisms underlying brain diseases.

AD is characterized by impaired neuronal inhibitory function or continuous overexcitement [58,59]. In the brains of Alzheimer’s disease patients, abnormal changes in recurrent connection strength, as one of the direct manifestations of AD neurodegenerative changes, reflect alterations in neuronal connections and information transfer efficiency. To simulate the evolution of AD, we altered the strength of excitatory neurons in our model. We increased recurrent connection strength ω to simulate disease advancement and decreased ω to represent theoretical intervention. The simulations replicated typical network dysfunctions in AD, such as reduced synchrony, while also offering a mechanistic explanation. That is, the abnormal rise in local circuit excitability can disrupt the coordinated dynamics of large-scale brain networks. This finding is particularly relevant given that reducing the excitability of excitatory neurons and increasing the inhibitory of inhibitory neurons have been proposed as potential therapeutic strategies for AD [60–62]. Our model provides strong computational evidence for the neuronal hyperexcitability hypothesis of AD, and demonstrates the application prospects in translational neuroscience..

### Limitations and future prospects

Although SI-BCM effectively simulates individualized brain dynamics, several limitations remain. Currently, the model relies solely on functional data, omitting the crucial roles of individualized structural connectivity and signal conduction delay, which limits its biological plausibility. Additionally, the use of genetic algorithms does not provide parameter confidence intervals, and assuming a fixed hemodynamic response function may overlook inter-individual differences. Future work will address these issues by introducing Bayesian inference for uncertainty quantification, integrating multimodal prior information, and estimating individualized HRF. Beyond these technical improvements, a primary future direction involves applying SI-BCM to press significant neuroscience challenges, such as uncovering task-induced brain mechanisms, developing computational biomarkers for brain diseases, and advancing personalized virtual brain models to predict cognitive decline and treatment effects. Furthermore, a valuable extension of our framework would involve moving beyond the single-subject analysis presented here to conduct comparative studies on sparse dynamic components across different individuals or cognitive states. This cross-subject and cross-state comparison is expected to reveal how transient brain dynamics vary with behavior, diagnosis, and individual traits.

## Conclusion

In this study, we propose a data-driven reverse engineering framework, SI-BCM, designed to elucidate the neural mechanisms and characteristics underlying intrinsic brain dynamics. The framework’s key innovation is its capability to derive stable spatiotemporal co-activation patterns directly from time series, employing these patterns as the foundational engine for whole-brain simulation. Our results demonstrate that capturing intrinsic brain activity through spatiotemporal interactions effectively simulates brain function at the individual level. Specifically, the model showed high accuracy across a range of metrics and exhibited strong generalization and stability across different sessions. It also captured pronounced individual differences in brain activity. The simulation results correlated with cognitive performance, suggesting that it has the potential to explain individual differences in cognitive function. Furthermore, we observed that it is possible to simulate the changing pattern of AD brain activity by increasing the model parametric recurrent connection strength. This also suggests that changed brain activity patterns in AD patients may be associated with the overactivity of excitatory neurons. This model not only provides an effective tool for simulating the spatiotemporal pattern of global intrinsic brain activity at the individual level, but also provides a solid foundation for further exploration of brain changes in the spatiotemporal patterns of brain diseases.

## Materials and methods

### Data and preprocessing

Data consisted of resting-state scans and structural data from 150 unrelated subjects in the HCP 1200 Subjects release [63]. The resting-state fMRI data utilized the HCP minimal preprocessing pipeline [63]. The fMRI pipelines include following steps: distortion correction, motion correction, registration in structural data, and conversion to gray-ordinates standard space [64]. The acquired time series maintained 14.4-minute durations (equivalent to 1200 samples with 0.72 s repetition time). Meanwhile, the processed data were parcellated into 148 regions based on the 2009 Destrieux parcellation [65]. This parcellation manner aligns with the macroscopic anatomy of gyri and sulci, enabling accurate identification of brain regions [66,67]. SC matrices were constructed using probabilistic tractography. Following the HCP dMRI steps, fiber tracking was performed using the MRtrix3 package [68]. Furthermore, we selected high-level cognition items from the HCP protocol, including fluid intelligence (Cognition Fluid, HCP: CogFluidComp_Unadj, mean±SD: 116.99 ± 11.62, range: 94.78-144.58), crystallized intelligence (Cognition Crystallized, HCP: CogCrystalComp_Unadj, mean±SD: 119.91 ± 9.19, range: 98.59-146.89), and averaged the normalized scores of both fluid and crystallized intelligence (Cognition Total, HCP: CogTotalComp_Unadj, mean±SD: 125.38 ± 14.74, range: 95.55-153.36). Further details can be found on the HCP website (https://db.humanconnectome.org/).

Similarly, we used 90 subjects (three cohorts composed of 30 HC, 30 MCI, and 30 AD) from the ADNI dataset (http://adni.loni.usc.edu). During this time, they completed the Montreal Cognitive Assessment (MoCA, mean±SD: 21.87 ± 6.11, range: 4–30). The preprocessing steps for these data were the same as described above. Among them, the time series of the ANDI dataset was 9.35 min long (187 samples with 3 s repetition time).

Through cortical-surface spatial congruence analysis, we classified 148 brain regions into the seven canonical resting-state networks (RSN) delineated by B. T. Yeo et al. [69]. These RSNs comprise the following functional systems: the frontoparietal control network (FPN), default mode network (DMN), limbic network (LIM), dorsal attention network (DAN), salience/ventral attention network (VAN), somatomotor network (SMN), and visual network (VIS).

### Stable individualized brain computing model

The study aims to infer the inherent spatiotemporal collaboration principle, namely the spatiotemporal co-activity pattern, from a preprocessed time series, and then demonstrate that this pattern can drive highly realistic brain dynamics. The model architecture, similar to a traditional dynamical model, employs a set of differential equations to describe the evolution of each brain region (see formula (1)). The framework comprises two primary stages: (1) the inverse inference of the intrinsic spatiotemporal co-activity pattern, and (2) the generation of dynamic signals based on this pattern.


$$\frac{dx_{i}(t)}{dt}=\theta \cdot x_{i}(t)+\varphi \left(x_{i}(t)\right)+\xi \left(W\right)\cdot X(t)+\eta_{i}(t)$$ (1)

where the spatiotemporal reconstruction information captured from brain activity is characterized by ξ(W). ξ(·) is a function for constructing stable spatiotemporal co-activity patterns based on the collection of connection weights W. $x_{i}(t)$ denote the neural activity of the i-th brain region at time t, with X(t) representing the activity vector across all regions. Among them, i=1, 2, ..., M represents the index of brain regions, and M is the total number of brain regions. The parameter θ characterizes the self-attenuation rate of neural activity. The nonlinear function ϕ(·) captures the local dynamics and output within the neural population. Random noise $\eta_{i}(t)$ is added to the i-th region, modeling stochastic fluctuations in neural signals.

#### Spatiotemporal co-activity pattern.

Firstly, we divided the observed activity ${\{x}_{i}(t),\;t=\text{1},...\text{T},i=\text{1},...,M\}$ into N fragments and estimated the strength of transmission between brain regions in each fragment (see formula (2)). By the fusion algorithm of the augmented Lagrange multipliers and principal component pursuit (PCP) [70], the shared information is extracted from these transmission strength matrices (see formula (3) and (4)). It is reconstructed into a stable and symmetric stable connectivity pattern that contains the intrinsic shared information of the brain. It is important to emphasize that this differs from FC. Here, we set the fragment length to 30 time points and the step size to 10 time points. The choice of parameters involved in constructing the spatiotemporal weight matrix is discussed in the supplementary material (Fig B in S1 Text).


$$\sum {\mathrm{\backslash nolimits}}_{ti}\left(n\right)=\frac{\sum {\mathrm{\backslash nolimits}}_{i,j=1}^{M}\left(x_{i}\left(t\right)-\overset{―}{x_{i}}\right)\left(x_{j}\left(t\right)-\overset{―}{x_{j}}\right)}{\sqrt{\sum {\mathrm{\backslash nolimits}}_{i,j=1}^{M}{\left(x_{i}\left(t\right)-\overset{―}{x_{i}}\right)}^{2}\sum {\mathrm{\backslash nolimits}}_{i,j=1}^{M}{\left(x_{j}\left(t\right)-\overset{―}{x_{j}}\right)}^{2}}},n=1,\ldots ,N,t\in segment\;n$$ (2)

$$\left[vec\left(\sum {\mathrm{\backslash nolimits}}_{ti}\left(1\right)\right),vec\left(\sum {\mathrm{\backslash nolimits}}_{ti}\left(2\right)\right),\ldots ,vec\left(\sum {\mathrm{\backslash nolimits}}_{ti}\left(N\right)\right)\right]=W=L_{n}+S_{n}$$ (3)

$$\left(\frac{\text{1}}{N}\sum_{n=\text{1}}^{N}L_{n}\right)\overset{\text{yields}}{\to }W_{L}$$ (4)

where $L_{n}=\left[l_{\text{1}},...,l_{N}\right]$ is the low-rank matrix with $\text{rank}\left(L_{n}\right)=r_{n}<min\left(N,\frac{M(M-\text{1})}{\text{2}}\right)$, $S_{n}=\left[s_{\text{1}},...,s_{N}\right]$ is a sparse matrix with a fraction s of non-zero entries. For the decomposition process, we consider solving the following convex optimization problem (see formula (5)) to optimize $L_{n}$ and $S_{n}$ [70]:


$$\begin{array}{c} \begin{array}{c} min\\ \left\{L_{n}\right\},\left\{S_{n}\right\} \end{array}\sum_{n=\text{1}}^{N}{\left\|L_{n}\right\|}_{*}+\lambda_{\text{1}}\sum_{n=\text{1}}^{N}{\left\|S_{n}\right\|}_{\text{1}}+\lambda_{\text{2}}\sum_{n=\text{1}}^{N}{\left\|Bvec\left(L_{n}\right)\right\|}_{\text{1}}\\ \text{s}.\text{t}.\;{\text{L}}_{\text{n}}+{\text{S}}_{\text{n}}=\text{W},\;\forall \text{n}=\text{1},...,\text{N} \end{array}$$ (5)

where the low-rank component ${\left\|L_{n}\right\|}_{*}=\sum_{e=\text{1}}^{\gamma_{n}}\sigma_{e}\left(L_{n}\right)$ captures stable connection pattern shared across time fragments and thus reflects the underlying. It represents the underlying structure of the inherent and persistent brain connectivity, revealing widespread interaction patterns between brain regions. The sparse component ${\left\|S_{n}\right\|}_{\text{1}}=\sum_{ij}\left|s_{nij}\right|$ captures instantaneous fluctuations in each time fragments that may arise from spontaneous neural events or measurement noise. A regularization parameter $\lambda_{\text{1}}>\text{0}$ controls the trade-off between these components, ensuring that the model remains both flexible and stable. When $\lambda_{\text{1}}$ is large, the model tends to attribute more dynamic changes to instantaneous fluctuations, potentially leading to information loss in the low-rank component. When $\lambda_{\text{1}}$ is relatively small, the model absorbs more time-varying connection information into the pattern, which is not conducive to forming stable connection patterns..

Additionally, we introduce a regularization term ${\left\|Bvec\left(L_{n}\right)\right\|}_{\text{1}}$ to promote homogeneity across individual connectivity profiles. This term penalizes deviations from a common connectivity pattern, thereby enhancing the model’s ability to capture shared neural dynamics while accommodating individual variability. Define a matrix $\text{B}=\text{D}⨂{\text{A}}_{{\text{M}}^{\text{2}}}$ where


$$\text{D}=(\begin{array}{ccc} \begin{array}{ccc} -\text{1} & \text{1} & \text{0}\\ \text{0} & -\text{1} & \text{1} \end{array} & \cdots & \begin{array}{c} \text{0}\\ \text{0} \end{array}\\ \vdots & \ddots & \vdots \\ \begin{array}{ccc} \text{0} & \text{0} & \text{0} \end{array} & \cdots & \text{1} \end{array})$$ (6)

is a first-order difference matrix and ${\text{A}}_{{\text{M}}^{\text{2}}}$ is a $M^{\text{2}}\times M^{\text{2}}$ identity matrix.

The optimization problem is solved using the alternating direction method of multipliers (ADMM) [71], an iterative algorithm well-suited for handling complex, non-closed-form solutions. ADMM alternates between minimizing the objective function with respect to each set of primal variables and updating the dual variables, ensuring convergence to an optimal solution. This approach allows us to efficiently reconstruct the stable connectivity pattern, providing a stable and accurate representation of brain dynamics.

#### Whole-brain dynamic simulation based on stable pattern.

We use the stable connectivity pattern to drive the whole-brain dynamics model, thereby generating simulated neural activities and BOLD signals. Here, we extend our computational framework (see formula (7) - (9)) to a dynamic mean field model for illustrative purposes. This model is optimized based on a simplified spiking neuronal network, capturing the essential dynamics of neural activity across whole brain [72]. The key of the model is that its connection weights are not based on SC, but rather on stable patterns derived through inverse inference.


$$\frac{\text{d}{\text{S}}_{\text{i}}(t)}{\text{dt}}=-\frac{S_{i}(t)}{\tau_{s}}+r\left(\text{1}-S_{i}(t)\right)H\left(z_{i}(t)\right)+\sigma \eta_{i}(t)$$ (7)

$$\text{H}\left({\text{z}}_{\text{i}}(t)\right)=\frac{\text{a}{\text{z}}_{\text{i}}(t)-\text{b}}{\text{1}-\text{exp}\left(-\text{d}\left(\text{a}{\text{z}}_{\text{i}}(t)-\text{b}\right)\right)}$$ (8)

$$z_{i}(t)=\omega JS_{i}(t)+GJ\sum_{j}{\zeta (W)}_{ij}S_{j}(t)+I_{\text{0}}$$ (9)

The model incorporates total input current ${\text{z}}_{\text{i}}$, population firing rates $\text{H}\left({\text{z}}_{\text{i}}\right)$, and synaptic gating variables $S_{i}$, which are dynamically influenced by the recurrent connection strength ω, the excitatory subcortical inputs $I_{\text{0}}$, and inter-regional information flow. The global constant G quantifies the relative contribution of inter-cortical information transfer to the target region (i-th), relative to the recurrent connection and subcortical inputs. The function ${\zeta (W)}_{ij}$ regulates the connection strength from region j to region i based on the stable spatiotemporal co-activity pattern W. $\tau_{s}$ is the synaptic time constant, and r is a coefficient related to the time constant. J is the synaptic coupling strength. Parameter values for the input-output function $\text{H}\left({\text{z}}_{\text{i}}\right)$ were set to be a = 270 (n/C), b = 108 (Hz), and d = 0.154 (s). $\eta_{i}(t)$ is uncorrelated standard Gaussian noise, and the noise amplitude is controlled by σ.Parameters are calibrated based on prior research [40,72], ensuring biophysical plausibility and computational efficiency.

For blood oxygen level dependent (BOLD) signal generation, simulated neural activities are integrated with the Balloon-Windkessel hemodynamic model [47], a biophysical model that transforms synaptic activity into a cascade of physiological responses. This model translates synaptic activity $S_{i}$ into changes in vasodilatory signals $Z_{i}$, blood flow$f_{i}$, volume $v_{i}$, and deoxyhemoglobin content $q_{i}$, ultimately yielding BOLD signal estimates. The underlying physiological relationships are mathematically represented through the following system of equations (10) - (14):


$$\dot{Z_{i}}=S_{i}-kZ_{i}-\gamma \left(f_{i}-\text{1}\right)$$ (10)

$$\dot{f_{i}}v=Z_{i}$$ (11)

$$\tau \dot{v_{i}}=f_{i}-{v_{i}}^{\frac{\text{1}}{\alpha }}$$ (12)

$$\tau \dot{q_{i}}=\frac{f_{i}}{\rho }\left[\text{1}-(\text{1}-\rho {)}^{\frac{\text{1}}{f_{i}}}\right]-{q_{i}v_{i}}^{\frac{\text{1}}{\alpha }-\text{1}}$$ (13)

$${BOLD}_{i}=V_{\text{0}}\left[k_{\text{1}}\left(\text{1}-q_{i}\right)+k_{\text{2}}\left(\text{1}-\frac{q_{i}}{v_{i}}\right)+k_{\text{3}}\left(\text{1}-v_{i}\right)\right]$$ (14)

The model’s kinetic parameters are set according to established literature [72–74], and simulations are performed using Euler’s integration method with a fine temporal resolution.

### Cost function and optimization procedure

Traditionally, the cost function used in studies is the correlation between empirical and simulated FC, which only considers the relatively stable coordination activities between different brain regions and ignores the instantaneous changes. To more accurately capture the dynamic interactions between brain regions, we developed a PSA matrix (see formula (15) and (16)). This matrix quantifies the dynamic associations by evaluating the structural similarity between the phase-space trajectories of different brain regions. To embed the time series of each brain region into a three-dimensional phase space, we employed the time-delay embedding method. Specifically, the calculation formula for PSA is as follows:


$${PS}_{i}={{[x}_{i}(t),\;{\;x}_{i}(t+\tau ),\;x_{i}(t+\text{2}\tau )]}^{\wedge }T,\;\;t=\text{1},\;\text{2},...,T-\text{2}\tau$$ (15)

$${PSA}_{ij}=\frac{\left(\text{2}\overset{―}{{PS}_{i}}\cdot \overset{―}{{PS}_{j}}+{\text{c}}_{\text{1}})(\text{2}\sigma_{{PS}_{i}{PS}_{j}}+{\text{c}}_{\text{2}}\right)}{\left({\overset{―}{{PS}_{i}}}^{\text{2}}+{\overset{―}{{PS}_{j}}}^{\text{2}}+{\text{c}}_{\text{1}})({\sigma_{{PS}_{i}}}^{\text{2}}+{\sigma_{{PS}_{j}}}^{\text{2}}+{\text{c}}_{\text{2}}\right)}$$ (16)

where ${PS}_{i}$ and ${PS}_{j}$ represent the phase space trajectory vector of the brain regions i and j, used to capture the dynamic characteristics of different regions. And the time delay parameter τ was selected to precede the first local minimum of the autocorrelation function, ensuring that the reconstructed phase space effectively captures the dynamic characteristics of the system without excessive correlation or redundancy. ${\left[\cdot \right]}^{\wedge }T$ indicates vector transpose. Specifically, the activity states of brain region i at three consecutive time points (t, t+τ, t+2τ) were combined into a three-dimensional phase space vector, where the evolving trajectory of these points over time t depicts the dynamic evolution of activity in this brain region. $\overset{―}{PS}$ and $\sigma_{PS}$ represent the mean and standard deviation of the corresponding term. $\sigma_{{PS}_{i}{PS}_{j}}$ represents the covariance between the phase spaces of region i and j, reflecting their level of coordinated activity. ${\sigma_{{PS}_{i}}}^{\text{2}}$ and ${\sigma_{{PS}_{j}}}^{\text{2}}$ denote the phase space variance of region i and region j, reflecting the degree of dispersion of activities in each region. ${\text{c}}_{\text{1}}$ and ${\text{c}}_{\text{2}}$ are constants that ensure stability when the denominator is zero. The formula is asymmetric concerning i and j due to its reliance on the specific order of the two trajectories for covariance calculation. This asymmetry allows PSA to capture directional information about dynamic interactions between brain regions, contrasting with the symmetric nature of the functional connectivity matrix.

We use genetic algorithms (GA) to adaptively optimize model parameters, including the recurrent connection strength ω, the excitatory subcortical input ${\text{I}}_{\text{0}}$, the global constant G, and the noise amplitude σ, to maximize the similarity between the simulated PSA matrix and the measured PSA matrix:


$$\text{PSAC}=\text{corr}\left({\text{PSA}}_{\text{emp}},{\text{PSA}}_{\text{sim}}\right)$$ (17)

Here, we chose the GA because we believe that the algorithm is a class of global optimization techniques well-suited for high-dimensional, non-linear solution spaces with potential local optima. Inspired by the process of natural selection, GA efficiently explore vast parameter spaces to identify the combination that maximizes the objective function. Unlike other algorithms, it is less prone to becoming trapped in local optima, a critical advantage when exploring parameters of complex neural dynamics models.

In order to avoid the phenomenon of overfitting, we divide a single data into two segments. The first half of the data is used for model construction and the second half is used for model fitting. For each parameter set, we performed multiple simulations, averaged the resulting PSA matrices, and compared them with the measured PSA to ensure the model’s activity closely similar to real dynamics (BOLD signals recorded in the dataset). This method not only enhances the model’s ability to capture the dynamic characteristics of the brain in terms of accuracy and reliability, but also improves the robustness and applicability of the model.

### Model performance evaluation

We used the SI-BCM with the Windkessel-Balloon hemodynamic model, to estimate spontaneous neural activity signals and their FC. The simulated BOLD signal underwent temporal decimation to 0.72-second resolution (TR = 720ms), generating 1,200 time points to match the HCP rs-fMRI. At the same time, to comprehensively evaluate our model’s simulation performance, we employed metrics measuring similarity, including signal-similar, global network-similar, and subnet-similar metrics.

#### Frequency and time domain analysis.

Frequency domain analysis was achieved by the use of the Fourier transform algorithm to convert the BOLD signal from a time-domain signal to a frequency-domain signal. Time domain analysis was calculated by Hurst Exponent, which was used to characterize the complexity of BOLD signals.

#### Dynamic property analysis.

Dynamical property analysis is conducted to evaluate the temporal evolution of brain states, which is crucial for capturing the intrinsic complexity of neural dynamics. Previous research has highlighted the importance of synchrony and metastability in reflecting the brain’s capacity for flexible information processing [75]. Segregation and integration metrics enable quantification of the brain’s ability to maintain specialized processing while facilitating information exchange [76]. The integrated state occurrence rate (ISOR) quantifies the prevalence of integrated states over time, and further provides insight into the prevalence of integrated network configurations over time [77].

#### Functional connectivity strength analysis.

Functional connectivity strength analysis is performed to directly evaluate the similarity between simulated and empirical FC patterns. This is crucial for validating the model’s ability to reproduce brain network organization. The correlation of edge FC strength is assessed by correlating the upper triangular elements of the model’s z-transformed FC matrix with empirical FC data. The correlation of node FC strength is computed through pairwise association analysis between mean FC values derived from the simulated and empirical data. For each brain region i, the mean FC value is calculated as the average of its FC strengths with all other regions. Higher correlation values indicate superior model performance [78].

#### Topological properties analysis.

To evaluate the model’s ability to capture the organizational principles underlying brain functional network architecture, several quantitative measures were employed, including global and local efficiency, characteristic path length, clustering coefficient, participation coefficient, and modularity. These analytical computations were performed utilizing the Brain Connectivity Toolbox (BCT, accessible at http://www.brain-connectivity-toolbox.net) [79]. Specifically, global efficiency (Eglob) represents the inverse of the shortest path length across network nodes, while local efficiency (Eloc) quantifies node-specific connectivity by calculating the inverse of the mean shortest path connecting a given node to its adjacent nodes. These reveal the information transmission capabilities of both global and local networks. The characteristic path length (L) refers to the shortest average path length between all possible node pairs, indicating the network’s information transmission speed. Network segregation was evaluated through the clustering coefficient (CC), which reflects the proportion of established connections among neighboring nodes. The participation coefficient (PC) was implemented to measure the diversity of a node’s intermodule connections, whereas modularity (Q) served as an indicator of the network’s potential decomposition into such clearly delineated groups, highlighting the compactness of the network structure [80].

#### The analysis of within- and between-network functional connectivity of each RSN.

The analysis of within- and between-network FC is aimed at evaluating the cohesion and integration of functional networks, which reflects the hierarchical organization of the brain’s functional architecture [75]. Within-network FC is computed by averaging Pearson correlations among time series of voxels within each RSN, capturing the integrity within specific regions. Between-network FC is evaluated by correlating the average time series of each RSN with those of other networks, reflecting the coordination between different sub-networks.

#### Stability analysis.

We assess the robustness of model outputs across repeated simulations using test-retest reliability, which is crucial for establishing the reliability of model-generated data. This metric is quantified by the intraclass correlation coefficient (ICC) [81]. This metric evaluates the consistency of measurements across repeated sessions, providing a measure of reliability [82]. ICC values are categorized to reflect the level of reliability, with higher values indicating greater stability. The ICC value can be calculated according to the following formula:


$$\text{ICC}=\frac{{\text{MS}}_{\text{R}}-{\text{MS}}_{\text{E}}}{{\text{MS}}_{\text{R}}+(\text{k}-\text{1}){\text{MS}}_{\text{E}}}$$ (18)

${\text{MS}}_{\text{R}}$ denotes the average variance across different subjects, ${MS}_{E}$ signifies the mean squared error attributed to unexplained variability, and k corresponds to the total count of repeated measurements within the experimental framework.

#### Statistical analysis.

Statistical analyses was conducted utilizing the Statistical Package for Social Science (SPSS, version 19.0), while computational evaluations of metrics were executed in MATLAB. Model performance was assessed across two established metrics: global and subnet-level analyses. For group-level comparisons, permutation t-tests were employed, with adjustments for multiple comparisons made through the Bonferroni correction (significance threshold: p < 0.05). All p-values reported in this study were computed for two-tailed tests. Relationships between simulated functional connectivity strength and cognitive behaviors, as well as associations between model parameters and cognitive behaviors, were investigated using Spearman’s correlation analysis. To minimize confounding effects, gender and age were considered irrelevant variables and regressed out during the analysis.

## Supporting information

S1 Text This file contais Supplementary Information S1 Text, including Fig A-E. **Fig A in S1 Text. Instability analysis of sparse components.** a) Schematic diagram of the low-rank component. b), c), d), e), and f) Schematic diagrams of the sparse components corresponding to five randomly selected time segments. g) Consistency analysis of low-rank matrices and sparse matrices in different time segments. h) Averaged edge FC value of real and simulated data for every time segments. i) The correlations between the empirical and simulated FC were calculated by using the low-rank component and the sparse component as connection weights in the model, respectively. **Fig B in S1 Text. The influence of parameters in constructing weight matrix on model results.** a) The model results constructed with different window lengths. Among them, the model result is the correlation between the real FC and the simulated FC. The signal at time point 200 is on the upper side, and the signal at time point 400 is on the lower side. b) The model results constructed with different steps. The signal at time point 200 is on the upper side, and the signal at time point 400 is on the lower side. c) The model results constructed with different signal length. d) The model results constructed with different brain regions. **Fig C in S1 Text. The optimal parameter distribution for the AD model. Fig D in S1 Text. Simulation analysis for SC-BCM based on Alzheimer’s disease.** a) Exploring the changes of dynamic property and topological property of brain activity by continuously increasing the value of the recurrent connection strength ω for the CN model. b) Exploring the changes of dynamic property and topological property of brain activity by continuously adjusting the value of the recurrent connection strength ω for the MCI model. c) Exploring the changes of dynamic property and topological property of brain activity by continuously decreasing the value of the recurrent connection strength ω for the AD model. **Fig E in S1 Text. Simulation performance analysis for SI-BCM based on Kuramoto model.** a) Comparison of real data and model simulations based on signal analysis metrics. b) Comparison of real data and model simulations based on global network metrics. c) The measurement of the simulation performance for different subnets. VIS: visual network, SMN: somatomotor network, DAN: dorsal attention network, VAN: salience/ventral attention network, LIM: limbic network, FPN: frontoparietal control network, DMN: default mode network. * p < 0.05, **p < 0.01, *** p < 0.001.
(DOC)
