# Supplementary material for: Stable individualized brain computing model informed by spatiotemporal co-activity patterns
Source: PLoS Comput Biol. 2025 Dec 19;21(12):e1013817. doi: 10.1371/journal.pcbi.1013817 (PMC12716749; doi:10.1371/journal.pcbi.1013817)
Supplement: S1 Text — Fig A in S1 Text. Instability analysis of sparse components. a) Schematic diagram of the low-rank component. b), c), d), e), and f) Schematic diagrams of the sparse components corresponding to five randomly selected time segments. g) Consistency analysis of low-rank matrices and sparse matrices in different time segments. h) Averaged edge FC value of real and simulated data for every time segments. i) The correlations between the empirical and simulated FC were calculated by using the low-rank component and the sparse component as connection weights in the model, respectively. Fig B in S1 Text. The influence of parameters in constructing weight matrix on model results. a) The model results constructed with different window lengths. Among them, the model result is the correlation between the real FC and the simulated FC. The signal at time point 200 is on the upper side, and the signal at time point 400 is on the lower side. b) The model results constructed with different steps. The signal at time point 200 is on the upper side, and the signal at time point 400 is on the lower side. c) The model results constructed with different signal length. d) The model results constructed with different brain regions. Fig C in S1 Text. The optimal parameter distribution for the AD model. Fig D in S1 Text. Simulation analysis for SC-BCM based on Alzheimer’s disease. a) Exploring the changes of dynamic property and topological property of brain activity by continuously increasing the value of the recurrent connection strength ω for the CN model. b) Exploring the changes of dynamic property and topological property of brain activity by continuously adjusting the value of the recurrent connection strength ω for the MCI model. c) Exploring the changes of dynamic property and topological property of brain activity by continuously decreasing the value of the recurrent connection strength ω for the AD model. Fig E in S1 Text. Simulation performance analysis for SI-BCM based on Kuramoto [file pcbi.1013817.s001.doc]

**Supplemental Information For:**

**Stable Individualized Brain Computing Model informed by Spatiotemporal Co-activity Patterns**

Supplementary Methods: Detailed Description of Modeling Approaches

**SC-BCM Model**

We adopted a large-scale biophysical model based on structural connectivity, which is an extension of the classical dynamic mean-field model. The neural activity $S_{i}(t)$ of each brain region $i$ is described by the following stochastic differential equation:

$$\frac{dS_{i}(t)}{\mathrm{dt}}=-\frac{S_{i}(t)}{\tau_{s}}+r\left( 1-S_{i}(t) \right)H\left( x_{i}(t) \right)+\sigma\eta_{i}\left( t \right)$$

$$H\left( x_{i} \right)=\frac{ax_{i}-b}{1-exp\left( -d\left( ax_{i}-b \right) \right)}$$

$$x_{i}=\omega JS_{i}(t)+GJ\sum_{j} C_{ij}S_{j}(t)+I_{0}$$

where $H\left( x_{i} \right)$ denotes the population firing-rate function, $x_{i}$ is the total input current. The parameter $\omega$ represents the recurrent connection strength, and $I_{0}$ denotes the excitatory subcortical input. $G$ is the global coupling strength, and $C_{ij}$ is the structural connectivity matrix derived from dMRI. $\sigma$ specifies the noise intensity. Next, the simulated neural activities were combined with the Balloon-Windkessel hemodynamic model to obtain the BOLD signals. Additionally, we optimized parameters $\omega$, $I_{0}$, $G$, and $\sigma$ using a genetic algorithm to maximize the correlation between simulated and empirical FC.

**MINDy Model**

MINDy is a data-driven nonlinear dynamical system model used to estimate individualized brain dynamic models from resting-state fMRI. The discrete-time dynamical system equation of this model is:

$$x_{t+\Delta t}-x_{t}=(W\psi_{\alpha}(x_{t})-Dx_{t}+\varepsilon_{t})\Delta t$$

$$\psi_{\alpha}(x_{t}):=\sqrt{\alpha^{2}+{(bx_{t}+0.5)}^{2}}-\sqrt{\alpha^{2}+{(bx_{t}-0.5)}^{2}}$$

where the connection weight matrix, $W$, identifies causal influence pathways between neural populations. The parameterized sigmoid transfer function, $\psi_{\alpha}(x_{t})$, describes the relationship between a population's local activity and its output to other brain regions. The diagonal decay matrix, $D$, indicates how quickly a neural population returns to its baseline state after firing. Each node's curvature parameter is denoted by $\alpha$, and $\varepsilon_{t}$ represents the process noise. The fixed global hyperparameter is $b$, set at 20/3 in this example. Initially, individual fMRI data are input into the model. The model then employs the stochastic gradient descent algorithm to automatically and iteratively optimize all parameters, aiming to minimize the error between its dynamic predictions and actual brain activity changes. This process includes a regularization constraint. Finally, weak random noise is injected to simulate spontaneous brain activity fluctuations, allowing the model to run freely according to its intrinsic dynamic equations.

Supplementary Results:

**Instability analysis of sparse components**

The core of this study lies in the reverse inference of a stable connection pattern from spatiotemporal dynamic interactions to drive the whole-brain model. To achieve this, we utilized a decomposition algorithm to separate the connection matrix of each time segment into two components: a shared low-rank component and a transient sparse component S_n_. Additionally, we performed supplementary analyses to confirm the selection of the low-rank component as the primary driver of the model. The results (Fig S1) indicated a high level of consistency in the low-rank matrices derived from the same individual across different time segments, as evidenced by a high intra-class correlation coefficient (ICC = 0.9521), affirming their representation of the inherent and stable collaborative brain architecture. In contrast, the sparse matrices {S_1_, S_2_, ..., S_N_} from consecutive time segments exhibited significant variability, with notably low structural similarity among them (average correlation coefficient ICC = 0.0416). This pronounced instability and lack of reproducibility suggested that S_n_ likely reflected transient neural fluctuations, measurement noise, or brief cognitive events not captured by the model, rather than serving as a sustainable and reliable computational substrate. Consequently, this study identified the low-rank component as the exclusive core connection weight of SI-BCM based on its stability, reproducibility, and potential for sharing, while proposing a detailed exploration of S_n_ as a prospective research avenue.


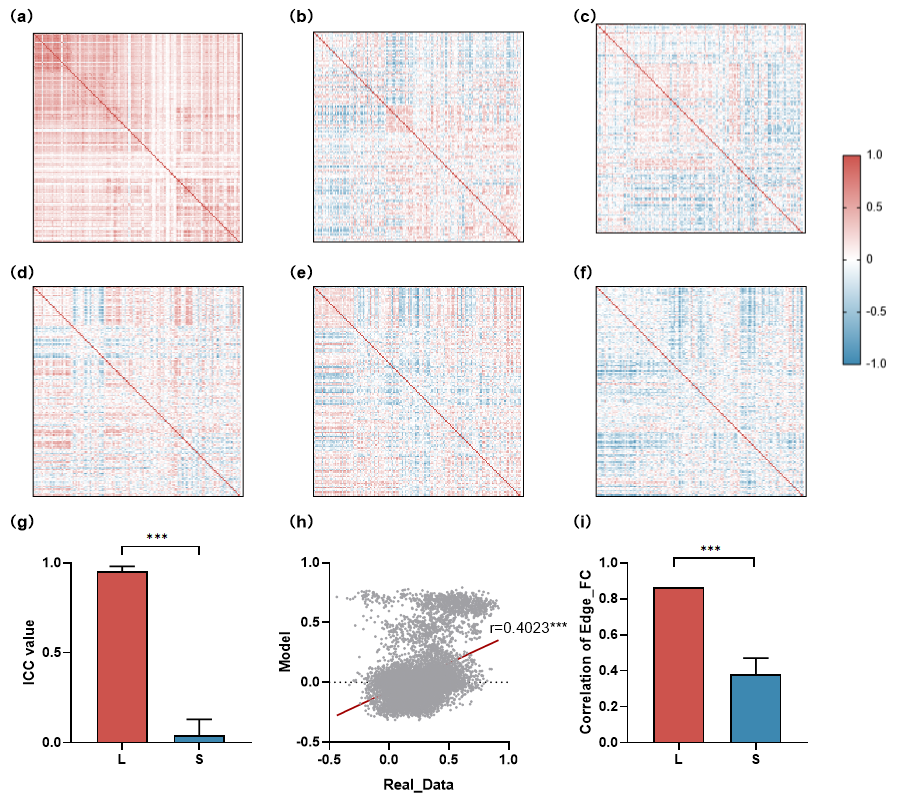


**Fig A. Instability analysis of sparse components.** a) Schematic diagram of the low-rank component. b), c), d), e), and f) Schematic diagrams of the sparse components corresponding to five randomly selected time segments. g) Consistency analysis of low-rank matrices and sparse matrices in different time segments. h) Averaged edge FC value of real and simulated data for every time segments. i) The correlations between the empirical and simulated FC were calculated by using the low-rank component and the sparse component as connection weights in the model, respectively.

**The impact of parameters in the construction of the weight matrix on model results**

In order to ensure that the constructed model can achieve the best accuracy, we also analyzed the parameters involved in the construction of the spatiotemporal weight matrix. Here, we used to control a single variable. Among them, when studying the effects of window length and step size, length of signal were taken at 200 and 400 time points, respectively. And when studying the effect of window length, the step size is set to 1 time point. This result indicates that the model is not affected by window length (Fig S2a). When studying the effect of step size, the window length was set to 30 time points. This result indicates that the model is also not affected by the step size (Fig S2b). When further studying the influence of signal length, the window length is set to 30 time points and the step size is set to 10 time points. It is evident from the Fig S2c that a relatively short length of signal will affect the accuracy of the model, but signals with a length exceeding 400 time points will not affect the model accuracy. Finally, when studying the effect of the number of brain regions, the window length was set to 30 time points, the step size was set to 10 time points, and the signal length was set to 1200 time points. Fig.S2d provides evidence that the model is not affected by the number of brain regions.


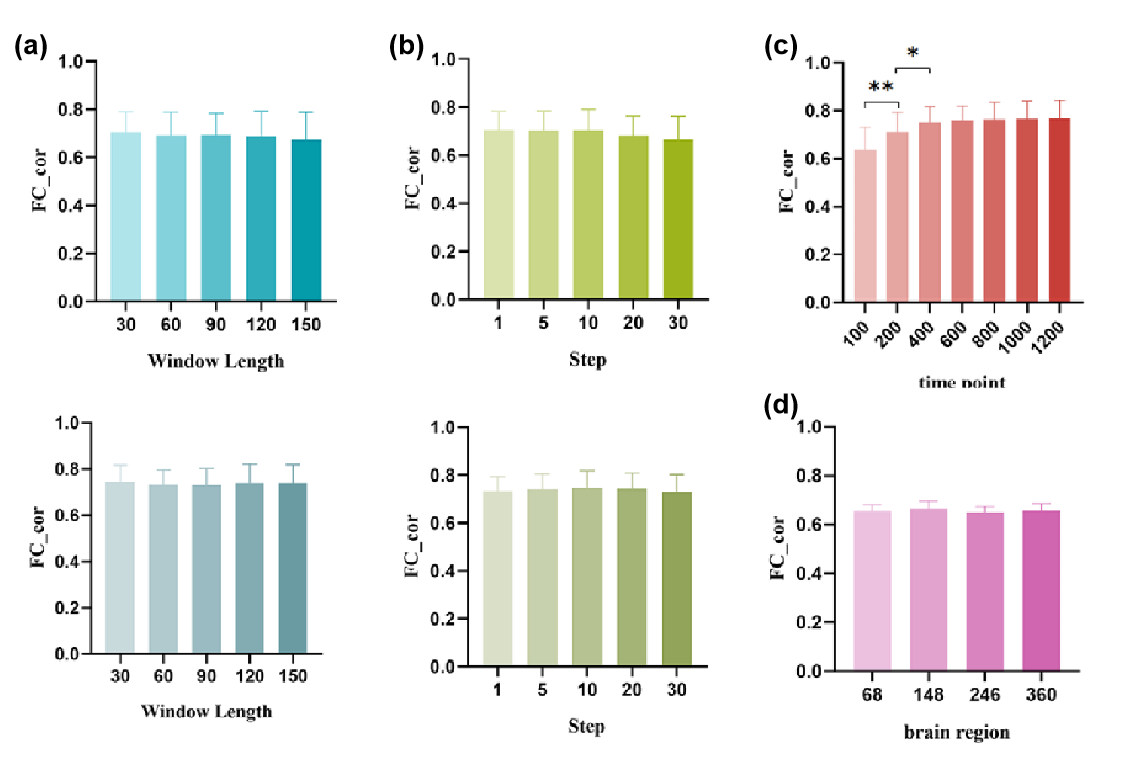


**Fig B. The influence of parameters in constructing weight matrix on model results.** a) The model results constructed with different window lengths. Among them, the model result is the correlation between the real FC and the simulated FC. The signal at time point 200 is on the upper side, and the signal at time point 400 is on the lower side. b) The model results constructed with different steps. The signal at time point 200 is on the upper side, and the signal at time point 400 is on the lower side. c) The model results constructed with different signal length. d) The model results constructed with different brain regions.

**AD parameters**

We constructed SI-BCM with optimal parameters for CN, MCI and AD, respectively, and briefly analysed the parameters. The results in Fig S3 show that the recurrent connection strength $\omega$ first increases and then decreases to normal levels. In addition, the noise $\sigma$ is also lower than normal.


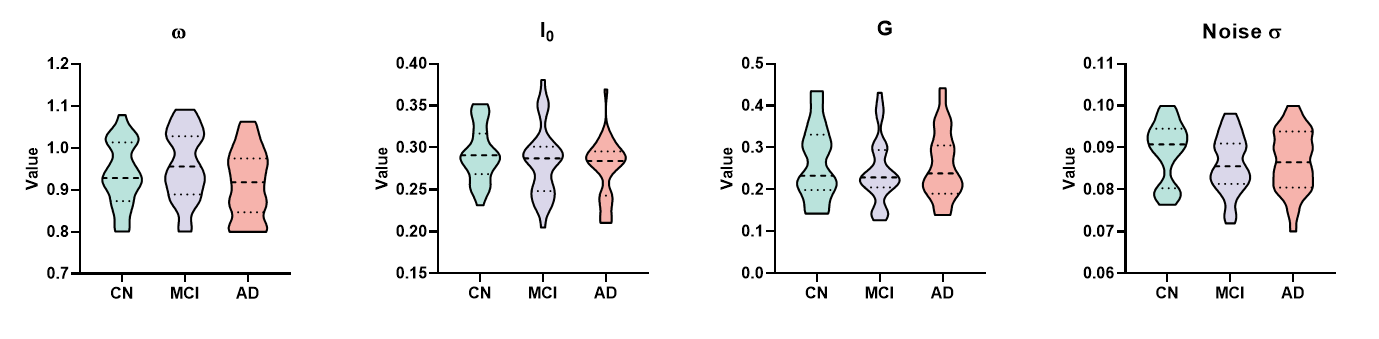


**Fig C. The optimal parameter distribution for the AD model.**

Besides, we adjusted the model parameters of SC-BCM to assess the effect of SC-BCM on AD disease evolution with the same methodology as in the main manuscript. From Fig S4, the values for the four indicators demonstrated a decreasing trend, but the capacity to capture these properties is not as effective as that of the SI-BCM.


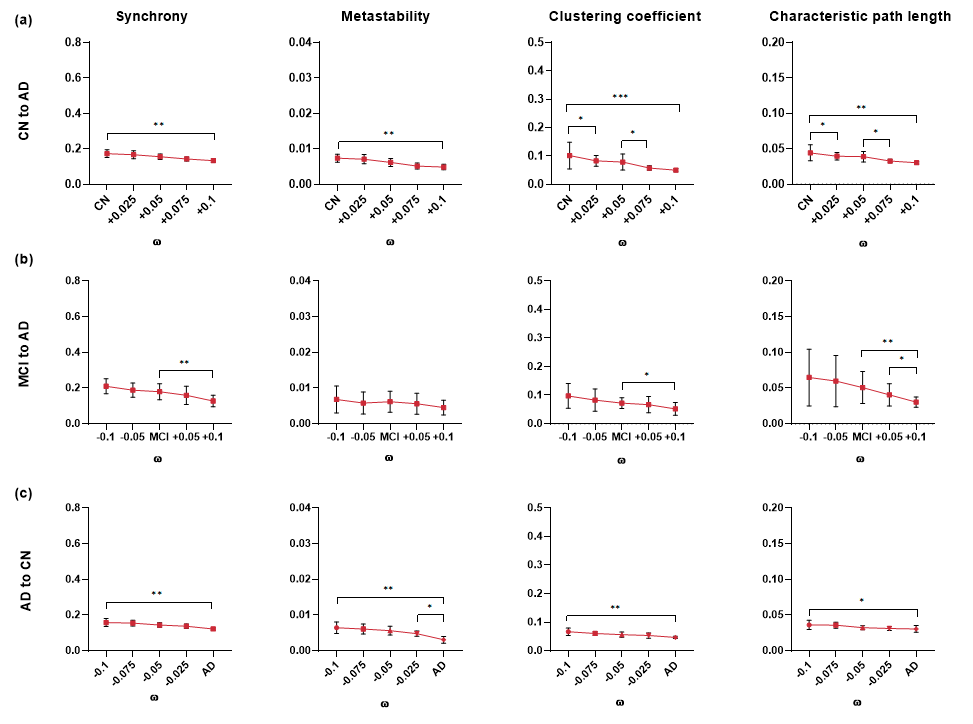


**Fig D. Simulation analysis for SC-BCM based on Alzheimer’s disease.** a) Exploring the changes of dynamic property and topological property of brain activity by continuously increasing the value of the recurrent connection strength $\omega$ for the CN model. b) Exploring the changes of dynamic property and topological property of brain activity by continuously adjusting the value of the recurrent connection strength $\omega$ for the MCI model. c) Exploring the changes of dynamic property and topological property of brain activity by continuously decreasing the value of the recurrent connection strength $\omega$ for the AD model.

**Kuramoto Model**

We also extend our computational framework to Kuramoto model for illustrative purposes. This model is optimized based on an oscillator(Cabral and Hugues et al., 2011), capturing the essential dynamics of neural activity across whole brain:

$$\frac{\text{d}\theta_{n}}{dt}=\omega_{0}+k\sum_{p=1}^{N} \zeta\left(W\right)\sin(\theta_{p}(t-\tau_{np})-\theta_{n}(t))+\eta_{n}(t),n=1,\ldots,N.$$

Where k is the global coupling strength which scales all connection strengths. $\tau_{\text{np}}$ is the time delay of interactions between brain regions n and p. The oscillators have intrinsic frequencies in the range of gamma. The time delay is proportional to the fiber length L of the connection between brain regions n and p such that $\tau_{\text{np}}=L_{np}\text{/}V=\langle\tau\rangle L_{np}/\langle L\rangle$. Delay of connectivity and distance matrix is fixed in their structures: the adjustable parameters are the global coupling strength $k$ and the mean delay $\langle\tau\rangle$. The terms $\eta_{\text{n}}\left(t\right)$, representing the noise of the local network to receive, correspond to Gaussian white noise with zero mean. Parameters are calibrated based on prior research(Cabral and Hugues et al., 2011; Vasa and Shanahan et al., 2015), ensuring biophysical plausibility and computational efficiency.

The results (Fig S5) show that our model has superior simulation results, with no significant differences found compared to the empirical observed data. Additionally, the SI-BCM based on Kuramoto model incorporates time delay, which allows the model to more accurately reflect fluctuations of synchrony in brain networks, consistent with the previously observed enhancing effect of time delay on network synchronization. Our findings demonstrate that SI-BCM with time delays maintains its efficiency and accuracy in simulating individual brain activity.


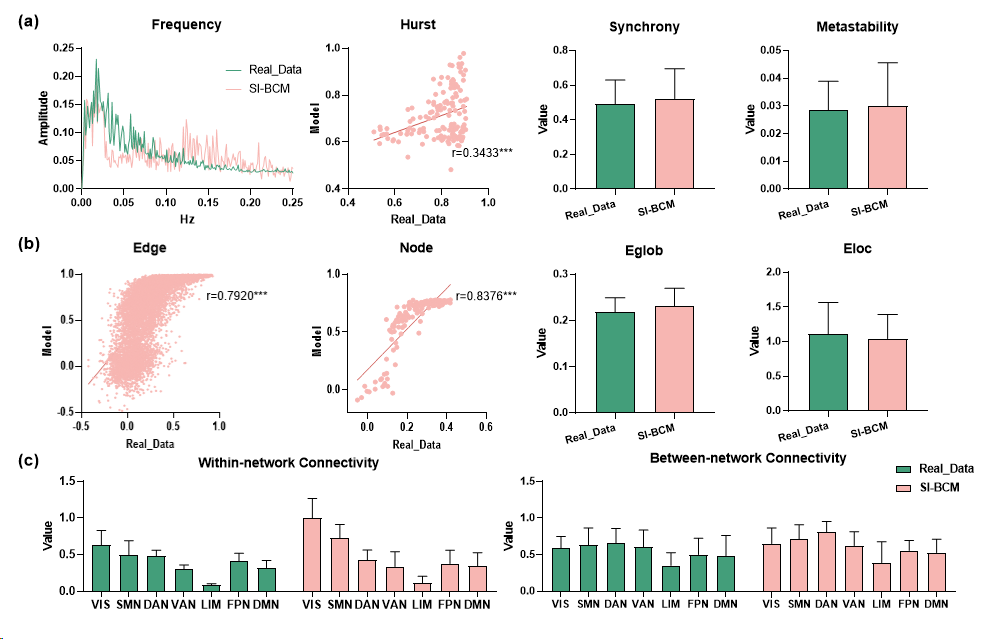


**Fig E. Simulation performance analysis for SI-BCM based on Kuramoto model.** a) Comparison of real data and model simulations based on signal analysis metrics. b) Comparison of real data and model simulations based on global network metrics. c) The measurement of the simulation performance for different subnets. VIS: visual network, SMN: somatomotor network, DAN: dorsal attention network, VAN: salience/ventral attention network, LIM: limbic network, FPN: frontoparietal control network, DMN: default mode network. * p<0.05, **p<0.01, *** p<0.001.
